# Clinical profile of probable cluster headache without ipsilateral autonomous symptoms

**DOI:** 10.1186/1129-2377-14-S1-P43

**Published:** 2013-02-21

**Authors:** N Imai

**Affiliations:** 1 Japanese Red Cross Shizuoka Hospital, Japan

## Introduction

Cluster headaches (CH), characterized by strictly unilateral pain localized in or around the eye and accompanied by ipsilateral autonomic features, are the most painful form of primary headache. Probable cluster headache (PCH) is a subtype of CH fulfilling all but one diagnostic criteria for it: for example, with no ipsilateral autonomic features. CH is associated with severe pain and has a considerable impact on social functioning and quality of life. Like CH, PCH without ipsilateral autonomic features (PCHWOIAF) may be associated with severe pain and considerable impact, but PCHWIAF has not been studied. The present study aimed to clarify the clinical profile of PCHWIAF.

## Methods

Seven patients who had been diagnosed with PCHWOIAF according to the 2nd edition of the International Classification of Headache Disorders were compared with 86 patients with CH. We collected data on laterality and location of headache, pain intensity, impact, additional features (sense of restlessness during the attacks, nausea, vomiting, photophobia and phonophobia), duration of attacks, and time of onset of attacks.

## Results

Pain occurred in the forehead, occipital regions, and vertex significantly more often in patients with PCHWOIAF than in patients with CH. There were no significant differences between patients with PCHWOIAF and CH in the mean age at first consultation, mean age of onset, the ratio of males to females, laterality of headache, pain intensity, impact, additional features, duration of attacks, and time of onset of attacks.

## Conclusion

The impact of PCHWOIAF is similar to that of CH. Patients with PCHWOIAF should receive the same level of treatment as patients with cluster headaches.
